# ViTSTR-Transducer: Cross-Attention-Free Vision Transformer Transducer for Scene Text Recognition

**DOI:** 10.3390/jimaging9120276

**Published:** 2023-12-13

**Authors:** Rina Buoy, Masakazu Iwamura, Sovila Srun, Koichi Kise

**Affiliations:** 1 Department of Core Informatics, Graduate School of Informatics, Osaka Metropolitan University, Osaka 599-8531, Japan; 2 Department of Information Technology Engineering, Faculty of Engineering, Royal University of Phnom Penh, Phnom Penh 12156, Cambodia

**Keywords:** vision transformer (ViT), scene text recognition (STR), cross-attention, RNN-T, autoregressive language model

## Abstract

Attention-based encoder–decoder scene text recognition (STR) architectures have been proven effective in recognizing text in the real world, thanks to their ability to learn an internal language model. Nevertheless, the cross-attention operation that is used to align visual and linguistic features during decoding is computationally expensive, especially in low-resource environments. To address this bottleneck, we propose a cross-attention-free STR framework that still learns a language model. The framework we propose is ViTSTR-Transducer, which draws inspiration from ViTSTR, a vision transformer (ViT)-based method designed for STR and the recurrent neural network transducer (RNN-T) initially introduced for speech recognition. The experimental results show that our ViTSTR-Transducer models outperform the baseline attention-based models in terms of the required decoding floating point operations (FLOPs) and latency while achieving a comparable level of recognition accuracy. Compared with the baseline context-free ViTSTR models, our proposed models achieve superior recognition accuracy. Furthermore, compared with the recent state-of-the-art (SOTA) methods, our proposed models deliver competitive results.

## 1. Introduction

Scene text recognition (STR) is an optical character recognition task that recognizes text in the real world [1]. STR remains an unsolved problem because of many factors, such as complex background, noise inference, and irregular deformations [1]. With recent advances in deep neural networks, many sophisticated deep-learning-based STR methods [2,3,4,5,6,7,8,9,10,11,12,13,14] have emerged. Among them, transformer decoder-based and attention-based methods [4,6,7,8,9,10,11,12,13,14] have demonstrated their efficacy in the field of STR. The transformer decoder-based methods comprise two main components: a visual encoder and a linguistic decoder. The visual encoder is responsible for extracting visual features and their spatial relationships. The linguistic decoder functioning as a language model focuses on capturing character-level dependencies. The interactions between the visual and linguistic features are modeled via cross-attention mechanisms that allow the decoder to access pertinent segments of the encoder’s representations to generate one-at-a-time predictions. Nevertheless, as the size of visual features and the number of predicted characters increase, the model complexity and memory requirements in the cross-attention layers also grow proportionally. Let *H* and *W* be the height and width of an input image, and let $H^{\prime }$ and $W^{\prime }$ be the height and width of the resulting feature maps in which $H^{\prime }$ and $W^{\prime }$ are $\frac{H}{S_{h}}$ and $\frac{W}{S_{w}}$ for strike factors, $S_{h}$ and $S_{w}$, respectively. For $T_{d}$ target characters, the cross-attention complexity is $O(H^{\prime }W^{\prime }T_{d}D)$ for *D* model dimension. This computational caveat diminishes the attractiveness of the transformer decoder-based methods for a long textline recognition task or in a low-resource setting.

RNN-T [15,16], a technique developed for speech recognition, is a language-aware method, but it does not employ any cross-attention mechanisms. Thus, compared with other language-aware methods, RNN-T exhibits faster inference. However, during training, RNN-T produces an output sequence that does not align with a target sequence and the true alignment between an input sequence and a target sequence is not known in advance. Thus, RNN-T needs to identify all possible alignments during training and this leads to a memory-intensive training process [17]. In addition, it was designed to process only one-dimensional (1D) visual feature sequences.

If a single alignment is known or imposed in advance, it is possible to remove the memory-intensive training bottleneck of handling all possible alignments. In this regard, ViTSTR [18] is a context-free encoder-only method that produces an output sequence aligning with a target sequence and, thus, does not require handling all possible alignments. By default, the encoder of ViTSTR is a two-dimensional (2D) vision transformer (ViT) feature extractor. However, ViTSTR cannot learn a language model that is beneficial in challenging scenarios like character occlusion. Thus, in comparison to the language-aware methods, ViTSTR exhibits lower recognition accuracy.

Inspired by ViTSTR and RNN-T, we propose a cross-attention-free STR framework called ViTSTR-Transducer, which learns a language model but without any high-complexity cross-attention mechanisms. Like ViTSTR, our proposed framework uses a pre-trained 2D ViT backbone to generate a patch-level feature sequence that aligns with a target sequence. Our proposed framework surpasses ViTSTR through the incorporation of a decoder with low complexity (i.e., no cross-attention mechanism) similar to RNN-T, but it does not require handling all possible alignments during training.

We validated the proposed ViTSTR-Transducer framework by using a standard STR pipeline by training on a collection of public synthetic datasets and subsequently finetuning on a collection of public labeled real datasets. The performance of our proposed models was evaluated on public benchmark datasets.

Our contributions can be summarized as follows:

1. We propose a cross-attention-free STR framework called ViTSTR-Transducer. Thus, our proposed framework has a constant decoding time with respect to the size of visual feature maps.
2. The analysis of inference time and accuracy indicates that our ViTSTR-Transducer models offer considerably lower latency while maintaining competitive recognition accuracy, compared with the baseline attention-based models. Compared with the baseline context-free ViTSTR models, our ViTSTR-Transducer models achieve superior recognition accuracy.
3. Compared with the state-of-the-art (SOTA) attention-based methods, our ViTSTR-Transducer models achieve competitive recognition accuracy.
4. The ablation results on the encoder’s backbone show that a ViT-based backbone, via its self-attention layers, allows the rearrangement of feature order to align with that of a target sequence.

## 2. Related Work

New Depending on the manner in which characters are generated and refined, STR methods can be classified as context-free, context-aware, and enhanced context-aware methods, while many recent context-aware and enhanced context-aware methods [4,6,7,8,9,10,11,12,13,14,19] have achieved SOTA recognition performance on benchmark datasets, they also entail high latency because of one-at-a-time decoding. These methods reply on high-complexity cross-attention mechanisms to align visual and linguistic features during decoding. In contrast, context-free methods [2,3,5,18,20] require low latency because of parallel decoding but yield sub-optimal recognition performance.

New Section 2.1 provides an overview of the context-free methods, followed by the context-aware methods in Section 2.2 and the enhanced context-aware methods in Section 2.3, respectively. Lastly, we discuss the recent advances in SOTA vision transformer-based STR methods.

### 2.1. Context-Free Methods

Context-free methods are characterized by their ability to generate subsequent outputs without relying on information from previous outputs. Introduced by Graves et al. [21], a connectionist temporal classification (CTC) is a context-free algorithm that estimates a total probability between an input sequence and an output sequence by marginalizing over all possible alignments [22,23]. Many CTC-based methods [2,3,5,20] follow a similar framework that comprises an optional rectification module, a visual feature extractor, a sequence modeler, and a decoder (CTC). Vision transformer for STR (ViTSTR) by Atienza [18] can be viewed as a CTC-based method that uses a pre-trained ViT model and imposes a single alignment. A major drawback of the CTC-based methods and ViTSTR is their strong assumption of conditional independence. Consequently, the CTC-based methods and ViTSTR do not learn an internal language model [23] during training that is beneficial in challenging scenarios like character occlusion. Nevertheless, it is possible to integrate an external language model to provide guidance during CTC training [24].

### 2.2. Context-Aware Methods

Context-aware methods are characterized by their ability to generate subsequent outputs based on information from previous outputs. Attention-based methods are context-aware or language-aware. The attention-based methods [4,6,9] predict one character at a time by selecting relevant parts of features using its hidden state. This conditional decoding enables an attention-based decoder to learn a language model implicitly. However, while an attention-based method achieves higher accuracy, it also comes with increased latency [25]. In the case of perspective or curved texts, 2D attention-based methods [7,8] were proposed. However, attending the entire visual feature maps during decoding increases the computation burden significantly.

Recurrent neural network transducer (RNN-T) by Graves [15,16], on the other hand, is an attention-free method but can acquire a language model. However, a major caveat of RNN-T is the intensive computation of a total probability for an input and output sequence pair by marginalizing over all possible alignments as the true alignment is not known in advance [17]. Nevertheless, Ngo et al. [26] experimented with an RNN-T-based offline handwritten text recognition for Japanese and Chinese scripts.

### 2.3. Enhanced Context-Aware Methods

The aforementioned context-aware methods utilize an autoregressive language model that strictly considers the context in a left-to-right manner. On the other hand, the enhanced context-aware methods improve the language modeling aspect of the context-aware methods by incorporating external language information, bidirectional context, or iterative refinement. Like the context-aware methods, the enhanced context-aware methods [10,11,12,13,14,19] still require cross-attention computations, the complexity of which is proportional to the size of visual feature maps and the number of decoded characters.

### 2.4. Vision Transformer-Based Methods

Inspired by the success of transformer networks [27] in natural language processing (NLP), Dosovitskiy et al. [28] introduced vision transformer (ViT) networks as a computation-friendly alternative to convolutional architectures. However, a major drawback of ViT is its reliance on large-scale training data due to the absence of inductive biases or priors. As a result, more data-efficient ViT architectures [29,30] have emerged. To capture multi-scale features, a hierarchical vision transformer network called Swin was proposed by Liu et al. [31], followed by an optimal searched architecture called S3 ViT network by Chen et al. [32]. The ViT architecture has rapidly gained popularity and has been widely embraced within STR methods. The ViT-based STR methods [18,33,34] have shown promising results.

## 3. Materials and Methods

### 3.1. Proposed Method

Building upon the inspiration from ViTSTR [18] and RNN-T [15,16], we introduce a cross-attention-free ViTSTR-Transducer framework for scene text recognition. This framework learns a language model without the need for cross-attention modules. Thus, ViTSTR-Transducer is a low-complexity and high-latency STR framework that does not sacrifice performance accuracy.

We begin by describing the building blocks of the proposed framework by detailing ViTSTR in Section 3.1.1, followed by RNN-T in Section 3.1.2. Lastly, the details of the proposed ViTSTR-Transducer framework are provided in Section 3.1.3.

#### 3.1.1. ViTSTR

**Figure 1:**
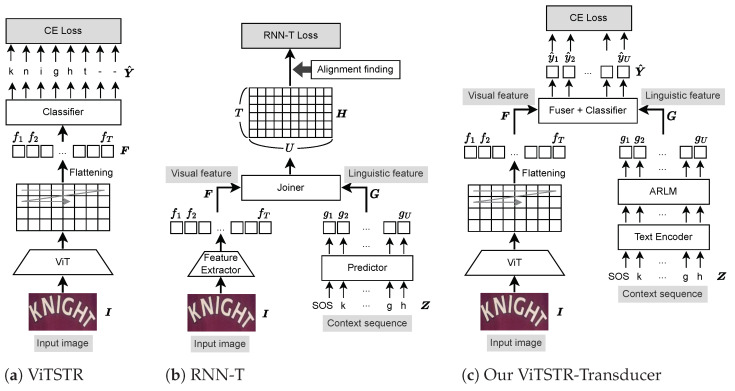
(**a**) ViTSTR architecture. ViTSTR uses a pre-trained ViT model to generate a flattened patch-level feature sequence that aligns with a target sequence. (**b**) RNN-T architecture. RNN-T models input–input and output–output dependencies without any cross-attention mechanisms. However, the RNN-T’s output does not align with a target sequence. (**c**) In our ViTSTR-Transducer, a ViTSTR-like encoder is used to encode an input image into F. The ARLM takes a context sequence, Z, and produces a contextual feature sequence, G, which is fused with F to output a class distribution sequence, $\hat{Y}$. *D* and *C* are the feature and class dimensions, respectively.

ViTSTR [18] is a context-free encoder-only architecture that is based on a pre-trained ViT backbone, as shown in Figure 1a. ViTSTR takes an input image, I, and generates a flattened patch-level feature sequence, F, that is forced to align with a training target sequence during training. $F=(f_{1},\ldots ,f_{T})$, $f_{i}\in R^{D}$, is expressed as

(1) F=ViT-Encoder(I)

where **ViT-Encoder** is a pre-trained ViT encoder, followed by a flattening operator. The flattening operator converts a 2D array of feature vectors into a 1D structure. Let $H^{\prime }$, $W^{\prime }$, and *D* be the height, width, and channel of the encoder’s 2D feature maps, respectively. Then, $T=H^{\prime }W^{\prime }$. The predicted character distribution sequence, $\hat{Y}=\left({\hat{y}}_{1},\ldots ,{\hat{y}}_{T}\right)$, ${\hat{y}}_{i}\in R^{C}$, is expressed as

(2) $$\begin{array}{c} \hat{Y}=\mathrm{SoftmaxClassifier}\left(F\right), \end{array}$$

where **SoftmaxClassifier** is a simple feed-forward network, followed by softmax normalization along class dimension and *C* is the number of class labels. A cross-entropy loss is calculated between the predicted character distribution, $\hat{Y}$, and the ground-truth text, Y. For example, in Figure 1a, $f_{1}$ and $f_{6}$ are trained to be the features of the letters *k* and *t*, respectively. During inference, ViTSTR employs a no-label token (i.e., a dash in Figure 1a) for indicating spaces or the end of decoding.

ViTSTR uniquely assumes that F aligns with Y. We discovered that the message-passing self-attention layers present in a ViT model allow for the rearrangement of feature order, making this assumption feasible. For example, the feature, $f_{1}$ in Figure 1a, belongs to the patch on the top left of the input image and is assigned to predict the first character regardless of whether the patch contains the first character or not. Even if the first character appears at the bottom right corner of the input image, the feature $f_{1}$ is learned to represent the bottom right region through self-attention layers. This is because the feature $f_{1}$ is determined by highly relevant features that contribute to the overall minimization of the cross-entropy loss.

#### 3.1.2. RNN-T

RNN-T [15,16] is a special form of the context-aware methods but it does not utilize any cross-attention mechanisms, as shown in Figure 1b. Alongside an encoder or a feature extractor, RNN-T employs a predictor and a joiner. The predictor network, functioning as an autoregressive (AR) left-to-right language model, solely relies on a context sequence. The joiner network is a simple feed-forward network that integrates visual and linguistic features from both the predictor and the encoder to generate character predictions. For an input image, I, and a context sequence, Z, as shown in Figure 1b, the outputs, $F=(f_{1},\ldots ,f_{T})$, $f_{i}\in R^{D}$, and $G=(g_{1},\ldots ,g_{U})$, $g_{i}\in R^{D}$, of the encoder and the predictor are expressed as

(3) $$\begin{array}{cc} F & =\mathrm{Encoder}(I) \end{array}$$

(4) $$\begin{array}{cc} G & =\mathrm{Predictor}(Z), \end{array}$$

where *T* and *U* are the lengths of a feature sequence and a context sequence, respectively. **Encoder** is usually a CNN-based 1D feature extractor. **Predictor** is usually an RNN language model.

Since F and G do not align with a target sequence and the true alignment is not known, RNN-T employs the joiner network to aggregate F and G by identifying all possible alignments. Let *C* be the number of class labels. The output, $H=(h_{1,1},\ldots ,h_{T,1},\ldots ,h_{T,U})$, $h_{i,j}\in R^{C}$, of the joiner is given by

(5) $$\begin{array}{c} H=\mathrm{Joiner}(F,G), \end{array}$$

where **Joiner** is a simple feed-forward network that outputs a three-dimensional tensor whose element is represented as $H_{ijk}=F_{ik}+G_{jk}$. For a given batch size, *B*, the space complexity of H is O(BTUC). By assuming that an alignment $a=(a_{1},a_{2},\cdots ,a_{K})$ is given and *K* is the length of a, the probability p(a) is given by

(6) $$\begin{array}{c} p\left(a\right)=\prod_{k=1}^{K}p_{k}\left(a_{k}\right), \end{array}$$

where $p_{k}\left(a_{k}\right)$ is a label probability of a at the *k*-th position. $p_{k}\left(a_{k}\right)$ is obtained from H [17].

During training, instead of computing p(a) over a single alignment, RNN-T computes a total probability, p(Y), over all possible alignments by using dynamic programming since there can be a large number of possible alignments. RNN-T loss is minimized by minimizing the negative logarithmic total probability, −logp(Y).

#### 3.1.3. ViTSTR-Transducer

Inspired by ViTSTR and RNN-T, ViTSTR-Transducer is a low-complexity autoregressive ViT-based STR framework but without any cross-attention mechanisms. The overall ViTSTR-Transducer framework is illustrated in Figure 1c. ViTSTR-Transducer uses a ViTSTR-like encoder to generate a flattened patch-level feature sequence. The pre-trained ViT model takes an input image, I, and outputs a 2D feature sequence that is flattened to form a 1D feature sequence, $F=(f_{1},\ldots ,f_{T})$. F is obtained by Equation (1). As we argued in Section 3.1.1, a flattened patch-level feature sequence of ViTSTR aligns with a target sequence. For instance, in Figure 1c, $f_{1}$ and $f_{6}$ are visual features corresponding to the letters *k* and *t*, respectively.

To integrate linguistic information, we employ an autoregressive left-to-right language model (ARLM) that utilizes preceding characters to predict the subsequent character. The ARLM takes a context sequence, Z. The ARLM can be transformer decoders or recurrent neural networks. The ARLM returns a contextual vector sequence, $G=(g_{1},\ldots ,g_{U})$, $g_{i}\in R^{D}$, that is aware of prior context. G is expressed as

(7) $$\begin{array}{c} G=\mathrm{ARLM}(\mathrm{Embedding}(Z)), \end{array}$$

where **Embedding** is a character embedding layer and *D* is the model dimension. *U* is the length of a context sequence. In actual implementation, F is truncated so that G and F share the same length (i.e., U=T).

To aggregate the visual and linguistic information, G and F are fused by weighted element-wise addition and followed by a softmax classifier to predict a class distribution sequence, $\hat{Y}=\left({\hat{y}}_{1},\ldots ,{\hat{y}}_{U}\right)$, ${\hat{y}}_{i}\in R^{C}$, where *C* is the number of class labels. $\hat{Y}$ is expressed as

(8) $$\begin{array}{cc} \alpha & =\sigma (\mathrm{Linear}(F\circ G)) \end{array}$$

(9) $$\begin{array}{cc} \hat{Y} & =\mathrm{SoftmaxClassifier}((1-\alpha )F+\alpha G), \end{array}$$

where ∘ is the Hadamard product and **Linear** is a linear projection layer. σ is a sigmoid activation function and $\alpha \in R^{D}$. **SoftmaxClassifier** is a simple feed-forward network, followed by softmax normalization along the class dimension.

When computing $\hat{Y}$, F and G align with each other. This means that $f_{1}$ and $g_{1}$ in Figure 1c are jointly responsible for predicting the letter *k*. This strict alignment assumption between F and G is not made when computing H in RNN-T and this is why it requires handling all possible alignments to estimate p(Y).

Since F and G align with a training target sequence, Y, $\hat{Y}$ also aligns with Y. Thus, the cross-entropy loss between a predicted class distribution sequence, $\hat{Y}$, and a training target sequence, Y, is given by

(10) $$\begin{array}{c} \mathrm{Loss}=\mathrm{CrossEntropy}(\hat{Y},Y), \end{array}$$

where **CrossEntropy** is a cross-entropy loss operator.

During inference, the context sequence, Z, is initialized with a start-of-sentence token, SOS, to compute G that is, together with F, used to estimate $\hat{Y}$ and a predicted character is sampled from ${\hat{y}}_{U}$, the last vector of $\hat{Y}$. The process is repeated until an end-of-sentence token, EOS, is reached.

### 3.2. Datasets

In this section, we describe the sources of our training, finetuning, and evaluation data, while the training data are synthetic, the finetuning and evaluation data are real labeled data.

#### 3.2.1. Public Synthetic Datasets

Because it may be impractical or impossible to obtain a large amount of real data for training a text recognition system, synthetic labeled data were utilized for this purpose. To expand the range of data available for training, three main synthetic datasets, namely MJSynth (MJ) [35], SynthText (ST) [36], and SynthAdd (SA) [8], were combined into a single training dataset. The total number of training samples used was 13.9 million images.

#### 3.2.2. Public Real Labeled Datasets

Due to the limited availability of real labeled data, we gathered information from multiple public data sources, including COCO [37], RCTW [38], Uber-Text [39], ArT [40], LSVT [41], ReCTS [42], OpenImages V5 [43], and TextOCR [44]. We utilized the refined versions of several datasets including COCO-Text, RCTW, Text, Uber-Text, ArT, LSVT, and ReCTS provided by Bautista and Atienza [10]. In total, there were 3.2 million real labeled images.

Consistent with standard evaluation procedures, our performance assessment data comprise the subsequent datasets: SVT [45], IIIT [46], IC13 [47], IC15 [48], SVTP [49], and CUTE80 [50]. For IC13 and IC15, images with fewer than three characters and non-alphanumeric characters were pruned. As a result, there remained 857 and 1811 images for IC13 and IC15, respectively.

### 3.3. Experiments

In this section, we present the experiment setup and implementation details.

#### 3.3.1. Experiment Setup

**Figure 2:**
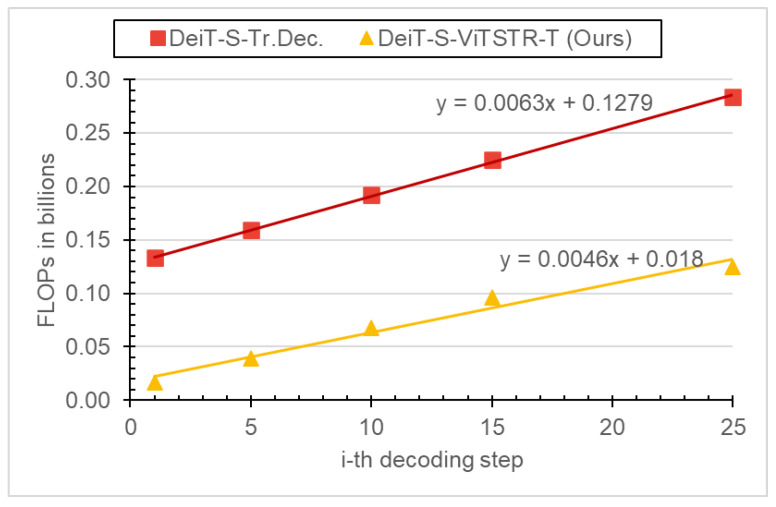
NewRequired FLOPs per each decoding step. ViTSTR-T: ViTSTR-Transducer. Tr.Dec.: transformer decoder. Best viewed in color.

To validate our ViTSTR-Transducer framework, we set up three cases: (1) the baseline ViTSTR models [18] (see Figure 1a), (2) the baseline transformer decoder-based models [7,34], and (3) our proposed ViTSTR-Transducer models (see Figure 1c). The baseline ViTSTR models follow the architecture by Atienza [18]. The baseline transformer decoder-based models use a standard transformer decoder [27] with cross-attention layers, while our ViTSTR-Transducer models use a modified transformer decoder without cross-attention layers. All models use a DeiT-Small backbone [30] as a 2D feature extractor since it is a good trade-off between model size and performance. Instead of RNN-T, the baseline transformer decoder-based models were employed to evaluate the impact of cross-attention mechanisms, compared with our cross-attention-free ViTSTR-Transducer models.

The specifications of the used DeiT-Small backbone are provided in Table 1 while the specifications of the baseline transformer-based decoder are presented in Table 2. The DeiT-Small backbone takes an input image of 32×128 pixels. The resulting feature maps are 8×16 and, thus, F is a sequence of 128 feature vectors (excluding class token vector), which is long enough to predict English words. Each feature vector, $f_{i}$, is in $R^{D}$, where D=384. The prediction covers only case-insensitive alphanumeric characters. In addition, three special characters (SOS, EOS, and PADDING) are included. Hence, C=39.

#### 3.3.2. Implementation Details

The models underwent a pre-training phase using synthetic datasets, which lasted for 15 full epochs. Within each epoch, the entire training data were used and a set of data augmentation techniques [10] was applied on each batch of 192 images. The training process utilized a cyclic learning schedule, with values ranging between $10^{-4}$ and $10^{-5}$, and a gradient clip of 50 was implemented.

After the training phase, the models underwent a finetuning process using real labeled datasets. This process continued for 50 full epochs and the same set of data augmentation techniques was applied. Similar to the pre-training phase, a cyclic learning schedule was used, with smaller values ranging between $10^{-5}$ and $10^{-6}$, and a gradient clip of 50 was applied during the finetuning process.

Rather than training the models with a combination of synthetic and real datasets all at once, dividing the training phase from the finetuning phase enables us to distinguish between weaknesses in the data and weaknesses in the models. This approach is particularly useful if the models struggle to generalize on the public benchmark datasets, as it allows us to identify the root cause of the issue more effectively. This was highlighted in a study by Liu et al. [51].

## 4. Results

In Section 4.1, we provide and evaluate the recognition accuracy and efficiency of our ViTSTR-Transducer models, compared with the baseline models. In Section 4.2, we present the ablation analyses of the complexities of the encoder and decoder. Lastly, in Section 4.3, we compare the recognition accuracy of our ViTSTR-Transducer models in comparison to the SOTA attention-based methods.

### 4.1. Recognition Accuracy and Efficiency Comparison with the Baseline Methods

We begin by evaluating the efficiency of the proposed ViTSTR-Transducer model (**DeiT-S-ViTSTR-T**) in comparison to the baseline methods (**DeiT-S-ViTSTR** and **DeiT-S-Tr.Dec.**), while the **DeiT-S-ViTSTR** model is encoder-only and context-free, our **DeiT-S-ViTSTR-T** and the baseline **DeiT-S-Tr.Dec.** models utilize an autoregressive left-to-right decoder. However, the decoder of the proposed model does not incorporate any cross-attention layers, the complexity of which is $O(H^{\prime }W^{\prime }T_{d}D)$.

Figure 2 compares the required FLOPs for each decoding step between our proposed **DeiT-S-ViTSTR-T** and the baseline **DeiT-S-Tr.Dec.** models. The figure illustrates the linear scaling of the required FLOPs for subsequent decoded characters. This linearly increasing pattern emerges because the autoregressive decoders of both our **DeiT-S-ViTSTR-T** and the baseline **DeiT-S-Tr.Dec.** models depend on previously decoded characters to predict the next one. At each decoding step, our **DeiT-S-ViTSTR-T** model required less than 44% of the FLOPs needed by the baseline **DeiT-S-Tr.Dec.** model. This improvement is attributed to the removal of cross-attention layers in the decoder of the proposed model. It should be noted that the baseline **DeiT-S-ViTSTR** model is not included in this figure because it is an encoder-only and context-free model that decodes all characters in parallel.

Table 3 presents the cumulative required FLOPs for an input image that is assumed to have the maximum number of 25 characters [18]. All models shared the same encoder’s FLOPs as they shared the same DeiT-Small backbone. The table suggests that our proposed **DeiT-S-ViTSTR-T** model requires only 37% of the cumulative FLOPs needed by the baseline **DeiT-S-Tr.Dec.** model to generate all characters one at a time for a given input image.

**Figure 3:**
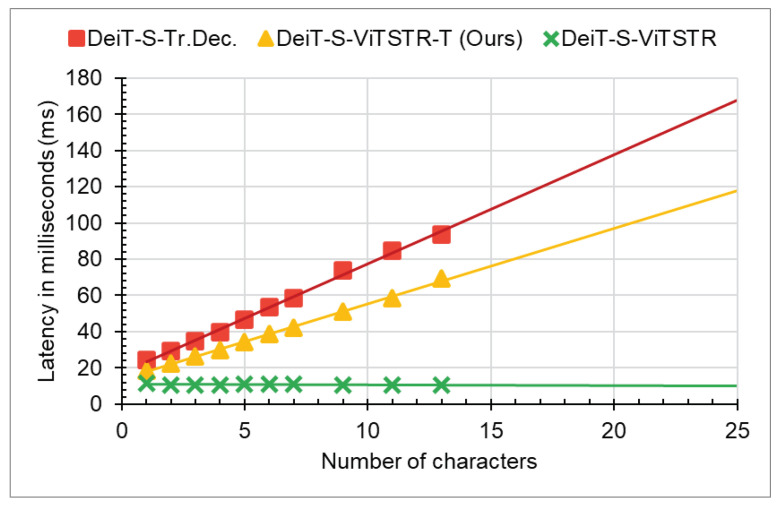
Inference time comparison on an RTX 2060 GPU. ViTSTR-T: ViTSTR-Transducer. Tr.Dec.: transformer decoder. Best viewed in color.

In terms of maximum latency (i.e., at 25 characters), Figure 3 suggests that our proposed model requires approximately 70% of the latency needed by the baseline **DeiT-S-Tr.Dec.** model. As shown in the same figure, the **DeiT-S-ViTSTR** model exhibited a constant latency regardless of the number of decoded characters. This is due to its parallel decoding nature, although it results in lower recognition accuracy.

To date, we have discussed the efficiency in terms of FLOPs and latency of our proposed model in comparison to the baseline models. Now, we focus on the recognition accuracy comparison. Table 4 compares the recognition accuracy for both synthetic (S) and real (R) training data cases. When trained on the synthetic training data case, as shown in Table 4a, our proposed model outperformed the baseline **DeiT-S-ViTSTR** model by achieving a total word recognition accuracy of 89.5% vs. 87.9%. However, the proposed model slightly underperformed, compared with the baseline **DeiT-S-Tr.Dec.** model that achieved a total accuracy of 91.1%.

When trained on the real training data, as shown in Table 4b, our proposed model outperformed the baseline **DeiT-S-ViTSTR** model by achieving a total word recognition accuracy of 95.3% vs. 94.4%. Additionally, its performance was competitive with the baseline **DeiT-S-Tr.Dec.** model that obtained a total accuracy of 95.9%.

In summary, our proposed method significantly reduces decoding latency while effectively maintaining a reasonably robust performance level, especially when trained on real training data.

### 4.2. Ablation Analyses of the Encoder and Decoder Complexities

The above experiments were conducted using the default Deit-Small [30] as a 2D backbone and a transformer-based decoder with three transformer decoder units, as provided in Table 2. To further evaluate the robustness of the proposed method, which is based on the assumption that the order of the visual features, F, aligns with the order of the linguistic features, G, we performed an ablation study on the encoder complexity by considering Deit-Tiny [30] and Deit-Medium [30] as backbones. Additionally, we assessed the decoder complexity by experimenting with the transformer-based decoders with one and five transformer decoder units (denoted as **DEC1** and **DEC5**, respectively).

As shown in Table 5, our proposed DeiT-*-ViTSTR-T (*: **T**, **S**, or **M**) models significantly outperformed the baseline DeiT-*-ViTSTR models regardless of the training data source and backbone. Compared with the baseline DeiT-*-Tr.Dec. models, our proposed models achieved comparable recognition accuracy. This highlights that the assumption that the order of the visual features, F, aligns with the order of the linguistic features, G, holds regardless of the backbone complexity. The table also indicates that increasing the backbone complexity results in a slight to moderate improvement in recognition accuracy. However, this improvement comes at the cost of a significant increase in model size, as demonstrated in Table 6. For instance, the **DeiT-M-ViTSTR-T** model requires twice as many parameters as our **DeiT-S-ViTSTR-T** model. Nevertheless, the total accuracy improvements were approximately 1.2% and 0.6% for the synthetic and real training data, respectively.

In addition, Table 5 also shows that as the encoder complexity increases (i.e., from Deit-T to DeiT-S to DeiT-M), the performance gaps between our proposed DeiT-*-ViTSTR-T and the baseline DeiT-*-Tr.Dec. models become smaller. This highlights the diminishing role of the cross-attention layers in the decoder in aligning the visual and linguistic features. Instead, this alignment is learned and achieved by the self-attention layers in the encoder side.

As demonstrated in Table 5, the impact of decoder complexity on the recognition accuracy was marginal, regardless of the training data source. However, increasing the decoder complexity directly resulted in significant increases in model size and inference time, as indicated in Table 6. Unlike the encoder, which processes only once for a given input image, the decoder iteratively processes until the decoding process is complete. Consequently, any latency increase per decoding step has a magnifying impact on the overall decoding latency, as illustrated in the case of the **DeiT-S-DEC5-ViTSTR-T** model, which requires up to 164.1 ms.

In summary, enhancing the encoder complexity can improve recognition accuracy but also results in a notable increase in model size. The impact of decoder complexity on recognition accuracy is marginal. However, increasing the decoder complexity can both enlarge the model size and significantly extend latency.

### 4.3. Recognition Accuracy Comparison with the SOTA Methods

In this section, we compare with the SOTA attention-based methods that employ cross-attention mechanisms to align visual and linguistic features during decoding. It should be highlighted that establishing fair comparisons with the SOTA methods is challenging due to the varying experimental conditions of each SOTA method. These conditions include factors, such as backbone architecture, data augmentation, pre-training strategy, training epochs, stopping criteria, and more.

Irrespective of the training data source, our proposed models achieved competitive recognition accuracy with the recent SOTA attention-based methods, as illustrated in Table 7. For instance, when trained on the synthetic data in Table 7a, our cross-attention-free **DeiT-S-ViTSTR-T** model obtained a total accuracy of 89.5% vs. 90.3% and 90.5% achieved by the SATRN [7] and SRN [14] models, respectively. In addition, utilizing the DeiT-Medium [30] as a backbone, the **DeiT-M-ViTSTR-T** model performed on par with the SOTA methods even without any cross-attention mechanisms. Similarly, when trained on the real training data in Table 7b, our proposed **DeiT-S-ViTSTR-T** model obtained a total accuracy of 95.3% vs. 95.1% and 95.7% achieved by the MAERec [52] and TRBA [10] models, respectively. Once again, the **DeiT-M-ViTSTR-T** model performed competitively with the SOTA methods.

Despite its smaller size, the **DeiT-T-ViTSTR-T** model showcased remarkable performance, as shown in Table 7. Notably, when trained on the real training data, it demonstrated comparable competitiveness with the DiG-ViT [34] models. This suggests that the **DeiT-T-ViTSTR-T** model strikes a harmonious balance between model size and recognition accuracy, making it a suitable choice for resource-constrained environments.

In summary, despite the absence of cross-attention mechanisms during decoding, the accuracy of our proposed models, particularly with real labeled data, competes effectively with that of the SOTA attention-based methods that depend on cross-attention mechanisms during decoding. Furthermore, the above experimental results further validate our proposed method’s primary assumption stating that the visual and linguistic features, denoted as F and G, align with the target character sequence. As a result, there is no need for cross-attention layers to align these two features.

## 5. Limitations and Future Work

We identify the following limitations of the proposed ViTSTR-Transducer framework.

- Because the decoder operates as a cross-attention-free autoregressive language model, it cannot effectively synthesize bidirectional linguistic information. This bidirectional context is valuable for handling occlusion cases and iterative refinement [10,11].
- In this study, ViT-based backbones have been proven to produce the visual features, F, that align with the linguistic features, G. However, further experiments are required to confirm whether this assumption is valid for a pure convolutional backbone or a hybrid convolutional transformer backbone.

Hence, our future work will address the first limitation by replacing the autoregressive language model with a masked language model that incorporates contextual information from both directions. Additionally, we will conduct experiments using a hybrid convolutional transformer architecture, instead of a vision transformer, to further validate our proposed ViTSTR-Transducer framework.

## 6. Conclusions

We present a cross-attention-free ViTSTR-Transducer framework that draws inspirations from a vision transformer for scene text recognition (ViTSTR) and recurrent neural network transducer (RNN-T). Our proposed framework does not need any cross-attention mechanisms that are present in mainstream attention-based encoder–decoder architectures during decoding. Nevertheless, our proposed framework can still acquire a language model. Despite having less computational demands and lower latency, our proposed ViTSTR-Transducer models achieve performance competitive to the recent SOTA attention-based STR methods. Our proposed models achieve a significant latency improvement over the baseline attention-based models while maintaining a comparable level of recognition accuracy.

## Figures and Tables

**Table 1:** Specifications of the DeiT-Small [30].

ViT Model	Params	FLOPs	Input Size	Model Dim, *D*	Feature Maps
DeiT-Small	28.5 M	3.2 B	32 × 128	384	8 × 16

**Table 2:** Specifications of the transformer-based decoder.

Parameters	Value
Model dimension	384
Decoder Stacks	3
Attention heads	8
Dropout	0.1
Feed-forward dimension	1536

**Table 3:** Cumulative FLOPs comparison in billions. Bold: highest. ViTSTR-T: ViTSTR-Transducer. Tr.Dec.: transformer decoder.

Model	Params	Encoder	Decoder
**DeiT-S-ViTSTR [18]**	21.4 M	**2.91**	0.00
**DeiT-S-Tr.Dec. [7,34]**	28.5 M	**2.91**	**5.25**
**DeiT-S-ViTSTR-T (Ours)**	**27.0 M**	**2.91**	1.95

**Table 4:** Word recognition accuracy comparison with the baseline methods. Bold: highest. ViTSTR-T: ViTSTR-Transducer. Tr.Dec.: transformer decoder.

(a) Methods trained on synthetic training data (S).							
**Method**	**IIIT**	**SVT**	**IC13**	**IC15**	**SVTP**	**CUTE**	**Total**
**DeiT-S-ViTSTR [18]**	92.2	90.0	93.8	79.7	82.2	82.9	87.9
**DeiT-S-Tr.Dec. [7,34]**	**94.4**	**92.3**	**95.5**	**84.5**	**87.1**	**88.9**	**91.1**
**DeiT-S-ViTSTR-T (Ours)**	92.7	92.1	**95.5**	82.3	85.7	84.0	89.5
(b) Methods trained on real labeled training data (R).							
**Method**	**IIIT**	**SVT**	**IC13**	**IC15**	**SVTP**	**CUTE**	**Total**
**DeiT-S-ViTSTR [18]**	97.8	94.9	97.6	88.8	89.3	94.1	94.4
**DeiT-S-Tr.Dec. [7,34]**	**98.6**	97.1	**97.9**	**90.6**	**93.6**	**97.9**	**95.9**
**DeiT-S-ViTSTR-T (Ours)**	98.2	**97.4**	97.7	89.6	92.4	95.8	95.3

**Table 5:** Ablation results of the encoder and decoder complexities. Bold: highest. ViTSTR-T: ViTSTR-Transducer. Tr.Dec.: transformer decoder. DeiT-T: DeiT-Tiny. DeiT-S: DeiT-Small. DeiT-M: DeiT-Medium. DEC1(5): one (five) unit(s) of transformer decoders.

(a) Methods trained on synthetic training data (S).							
**Method**	**IIIT**	**SVT**	**IC13**	**IC15**	**SVTP**	**CUTE**	**Total**
**DeiT-T-ViTSTR [18]**	90.2	88.3	90.4	75.9	78.4	79.4	85.1
**DeiT-T-Tr.Dec. [7,34]**	92.6	90.9	94.6	81.3	84.3	82.9	88.9
**DeiT-T-ViTSTR-T**	92.2	89.6	94.0	80.5	82.9	82.6	88.2
**DeiT-M-ViTSTR [18]**	93.8	90.7	94.4	81.8	84.7	85.4	89.5
**DeiT-M-Tr.Dec. [7,34]**	**94.9**	92.7	**96.0**	**84.8**	**88.2**	**88.2**	**91.5**
**DeiT-M-ViTSTR-T**	93.9	**93.5**	95.4	83.5	87.6	87.5	90.7
**DeiT-S-DEC1-ViTSTR-T**	93.2	91.5	95.4	82.4	86.2	84.7	89.7
**DeiT-S-DEC5-ViTSTR-T**	93.2	91.8	95.5	82.8	87.3	84.0	90.0
**DeiT-S-ViTSTR-T (Ours)**	92.7	92.1	95.5	82.3	85.7	84.0	89.5
(b) Methods trained on real labeled training data (R).							
**Method**	**IIIT**	**SVT**	**IC13**	**IC15**	**SVTP**	**CUTE**	**Total**
**DeiT-T-ViTSTR [18]**	96.2	93.5	96.3	86.7	86.4	92.3	92.6
**DeiT-T-Tr.Dec. [7,34]**	97.7	95.7	97.3	88.8	91.3	95.5	94.7
**DeiT-T-ViTSTR-T**	97.4	95.8	97.5	88.7	90.5	96.2	94.5
**DeiT-M-ViTSTR [18]**	98.2	96.9	97.8	89.0	92.9	97.6	95.3
**DeiT-M-Tr.Dec. [7,34]**	**98.6**	**97.5**	**98.6**	**91.0**	**94.0**	96.9	**96.2**
**DeiT-M-ViTSTR-T**	98.3	97.4	97.9	90.8	93.5	**97.9**	95.9
**DeiT-S-DEC1-ViTSTR-T**	98.1	96.8	97.9	89.8	93.5	96.2	95.4
**DeiT-S-DEC5-ViTSTR-T**	97.8	96.6	98.3	90.1	92.9	97.2	95.4
**DeiT-S-ViTSTR-T (Ours)**	98.2	97.4	97.7	89.6	92.4	95.8	95.3

**Table 6:** Model size and maximum inference time comparison. Bold: highest. ViTSTR-T: ViTSTR-Transducer. Tr.Dec.: transformer decoder. Bold: highest. DeiT-T: DeiT-Tiny. DeiT-S: DeiT-Small. DeiT-M: DeiT-Medium. DEC1(5): one (five) unit(s) of transformer decoders.

Method	Params	Time (ms)
**DeiT-T-ViTSTR [18]**	5.4 M	10.2
**DeiT-T-Tr.Dec. [7,34]**	7.2 M	151.3
**DeiT-T-ViTSTR-T**	6.9 M	110.9
**DeiT-M-ViTSTR [18]**	40.0 M	11.3
**DeiT-M-Tr.Dec. [7,34]**	50.6 M	160.7
**DeiT-M-ViTSTR-T**	**48.0 M**	112.0
**DeiT-S-DEC1-ViTSTR-T**	23.5 M	63.3
**DeiT-S-DEC5-ViTSTR-T**	30.6 M	**164.1**
**DeiT-S-ViTSTR-T (Ours)**	27.0 M	118.0

**Table 7:** Word recognition accuracy (%) comparison with the SOTA attention-based methods. ViTSTR-T: ViTSTR-Transducer. Bold: highest.

(a) Methods trained on synthetic training data.							
**Method**	**IIIT**	**SVT**	**IC13**	**IC15**	**SVTP**	**CUTE**	**Total**
ASTER [9]	93.4	**93.6**	91.8	76.1	78.5	79.5	87.1
NRTR [53]	93.7	90.2	93.6	74.5	78.3	86.1	87.0
SAR [8]	91.3	84.7	91.2	70.7	76.9	83.0	84.1
SEED [12]	93.8	89.6	92.8	80.0	81.4	83.6	88.4
SRN [14]	94.8	91.5	**95.5**	82.7	85.1	87.8	**90.5**
SATRN [7]	94.7	92.1	94.2	82.1	86.4	87.6	90.3
RCEED [54]	**94.9**	91.8	94.7	82.2	83.6	**91.7**	90.4
PerSec-ViT [33]	88.1	86.8	94.2	73.6	77.7	72.7	83.8
**DeiT-T-ViTSTR-T (Ours)**	92.2	89.6	94.0	80.5	82.9	82.6	88.2
**DeiT-M-ViTSTR-T (Ours)**	93.9	93.5	95.4	**83.5**	**87.6**	87.5	**90.7**
**DeiT-S-ViTSTR-T (Ours)**	92.7	92.1	**95.5**	82.3	85.7	84.0	89.5
(b) Methods trained on real labeled training data.							
**Method**	**IIIT**	**SVT**	**IC13**	**IC15**	**SVTP**	**CUTE**	**Total**
TRBA [55]	94.8	91.3	94.0	80.6	82.7	88.1	89.6
DiG-ViT-tiny [34]	96.4	94.4	96.2	87.4	90.2	94.1	93.4
DiG-ViT-small [34]	97.7	96.1	97.3	88.6	91.6	96.2	94.7
DiG-ViT-base [34]	97.6	96.5	97.6	88.9	92.9	96.5	94.9
TRBA [10]	**98.6**	97.0	97.6	89.8	**93.7**	97.7	95.7
MAERec (pre-training) [52]	98.0	96.8	97.6	87.1	93.2	**97.9**	95.1
**DeiT-T-ViTSTR-T (Ours)**	97.4	95.8	97.5	88.7	90.5	96.2	94.5
**DeiT-M-ViTSTR-T (Ours)**	98.3	**97.4**	**97.9**	**90.8**	93.5	**97.9**	**95.9**
**DeiT-S-ViTSTR-T (Ours)**	98.2	**97.4**	97.7	89.6	92.4	95.8	95.3

## Data Availability

Available upon request.

## References

[1] Chen X., Jin L., Zhu Y., Luo C., Wang T. (2021). Text recognition in the wild. ACM Comput. Surv..

[2] Wang J., Hu X. Gated Recurrent Convolution Neural Network for OCR. Proceedings of the 31st International Conference on Neural Information Processing Systems.

[3] Borisyuk F., Gordo A., Sivakumar V. Rosetta: Large scale system for text detection and recognition in images. Proceedings of the 24th ACM SIGKDD International Conference on Knowledge Discovery and Data Mining.

[4] Shi B., Wang X., Lyu P., Yao C., Bai X. Robust scene text recognition with automatic rectification. Proceedings of the 2016 IEEE Conference on Computer Vision and Pattern Recognition (CVPR).

[5] Shi B., Bai X., Yao C. (2017). An end-to-end trainable neural network for image-based sequence recognition and its application to scene text recognition. IEEE Trans. Pattern Anal. Mach. Intell..

[6] Lee C., Osindero S. Recursive recurrent nets with attention modeling for OCR in the wild. Proceedings of the 2016 IEEE Conference on Computer Vision and Pattern Recognition (CVPR).

[7] Lee J., Park S., Baek J., Oh S., Kim S., Lee H. On recognizing texts of arbitrary shapes with 2D self-attention. Proceedings of the 2020 IEEE/CVF Conference on Computer Vision and Pattern Recognition Workshops (CVPRW).

[8] Li H., Wang P., Shen C., Zhang G. Show, attend and read: A simple and strong baseline for irregular text recognition. Proceedings of the AAAI Conference on Artificial Intelligence.

[9] Shi B., Yang M., Wang X., Lyu P., Yao C., Bai X. (2019). Aster: An attentional scene text recognizer with flexible rectification. IEEE Trans. Pattern Anal. Mach. Intell..

[10] Bautista D., Atienza R. (2022). Scene text recognition with permuted autoregressive sequence models. Computer Vision—ECCV 2022, Proceedings of the 17th European Conference, Tel Aviv, Israel, 23–27 October 2022.

[11] Fang S., Xie H., Wang Y., Mao Z., Zhang Y. Read like humans: Autonomous, bidirectional and iterative language modeling for scene text recognition. Proceedings of the 2021 IEEE/CVF Conference on Computer Vision and Pattern Recognition (CVPR).

[12] Qiao Z., Zhou Y., Yang D., Zhou Y., Wang W. Seed: Semantics enhanced encoder–decoder framework for scene text recognition. Proceedings of the 2020 IEEE/CVF Conference on Computer Vision and Pattern Recognition (CVPR).

[13] Wang Y., Xie H., Fang S., Wang J., Zhu S., Zhang Y. From Two to One: A New Scene Text Recognizer with Visual Language Modeling Network. Proceedings of the 2021 IEEE/CVF International Conference on Computer Vision, ICCV 2021.

[14] Yu D., Li X., Zhang C., Liu T., Han J., Liu J., Ding E. Towards accurate scene text recognition with Semantic Reasoning Networks. Proceedings of the 2020 IEEE/CVF Conference on Computer Vision and Pattern Recognition (CVPR).

[15] Graves A. (2012). Sequence transduction with recurrent neural networks. arXiv.

[16] Graves A., Mohamed A., Hinton G. Speech recognition with deep recurrent neural networks. Proceedings of the 2013 IEEE International Conference on Acoustics, Speech Furthermore, Signal Processing.

[17] Lugosch L. Sequence-to-Sequence Learning with Transducers. November 2020. https://lorenlugosch.github.io/posts/2020/11/transducer/.

[18] Atienza R. Vision transformer for fast and efficient scene text recognition. Proceedings of the Document Analysis and Recognition—ICDAR 2021: 16th International Conference.

[19] Bhunia A., Sain A., Kumar A., Ghose S., Nath Chowdhury P., Song Y. Joint Visual Semantic Reasoning: Multi-Stage decoder for text recognition. Proceedings of the 2021 IEEE/CVF International Conference on Computer Vision (ICCV).

[20] Liu W., Chen C., Wong K., Su Z., Han J. Star-net: A spatial attention residue network for scene text recognition. Proceedings of the British Machine Vision Conference 2016.

[21] Graves A., Fernández S., Gomez F., Schmidhuber J. Connectionist Temporal Classification. Proceedings of the 23rd International Conference on Machine Learning—ICML ’06.

[22] Hannun A. (2017). Sequence Modeling with CTC. Distill.

[23] Jurafsky D., Martin J. (2009). Speech and Language Processing: An Introduction to Natural Language Processing, Computational Linguistics, and Speech Recognition.

[24] Diaz D., Qin S., Ingle R., Fujii Y., Bissacco A. (2021). Rethinking Text Line Recognition Models. arXiv.

[25] Baek J., Kim G., Lee J., Park S., Han D., Yun S., Oh S., Lee H. What is wrong with scene text recognition model comparisons? Dataset and model analysis. Proceedings of the 2019 IEEE/CVF International Conference on Computer Vision (ICCV).

[26] Ngo T., Nguyen H., Ly N., Nakagawa M. Recurrent neural network transducer for Japanese and Chinese offline handwritten text recognition. Proceedings of the Document Analysis and Recognition—ICDAR 2021 Workshops.

[27] Vaswani A., Shazeer N., Parmar N., Uszkoreit J., Jones L., Gomez A., Kaiser L., Polosukhin I. Attention is All you Need. Proceedings of the Advances in Neural Information Processing Systems.

[28] Dosovitskiy A., Beyer L., Kolesnikov A., Weissenborn D., Zhai X., Unterthiner T., Dehghani M., Minderer M., Heigold G., Gelly S. (2021). An Image is Worth 16 × 16 Words: Transformers for Image Recognition at Scale. arXiv.

[29] Touvron H., Cord M., Sablayrolles A., Synnaeve G., J’egou H. Going deeper with Image Transformers. Proceedings of the 2021 IEEE/CVF International Conference on Computer Vision (ICCV).

[30] Touvron H., Cord M., Jegou H. (2022). DeiT III: Revenge of the ViT. arXiv.

[31] Liu Z., Lin Y., Cao Y., Hu H., Wei Y., Zhang Z., Lin S., Guo B. Swin Transformer: Hierarchical Vision Transformer using Shifted Windows. Proceedings of the 2021 IEEE/CVF International Conference on Computer Vision, ICCV 2021.

[32] Chen M., Wu K., Ni B., Peng H., Liu B., Fu J., Chao H., Ling H. Searching the Search Space of Vision Transformer. Proceedings of the Advances in Neural Information Processing Systems 34: Annual Conference on Neural Information Processing Systems 2021, NeurIPS 2021.

[33] Liu H., Wang B., Bao Z., Xue M., Kang S., Jiang D., Liu Y., Ren B. Perceiving stroke-semantic context: Hierarchical contrastive learning for robust scene text recognition. Proceedings of the AAAI Conference on Artificial Intelligence.

[34] Yang M., Liao M., Lu P., Wang J., Zhu S., Luo H., Tian Q., Bai X. Reading and writing: Discriminative and generative modeling for self-supervised text recognition. Proceedings of the 30th ACM International Conference on Multimedia.

[35] Jaderberg M., Simonyan K., Vedaldi A., Zisserman A. (2014). Synthetic Data and Artificial Neural Networks for Natural Scene Text Recognition. arXiv.

[36] Gupta A., Vedaldi A., Zisserman A. Synthetic data for text localisation in natural images. Proceedings of the 2016 IEEE Conference on Computer Vision and Pattern Recognition (CVPR).

[37] Veit A., Matera T., Neumann L., Matas J., Belongie S. (2016). COCO-Text: Dataset and Benchmark for Text Detection and Recognition in Natural Images. arXiv.

[38] Shi B., Yao C., Liao M., Yang M., Xu P., Cui L., Belongie S., Lu S., Bai X. ICDAR2017 competition on reading Chinese text in the wild (RCTW-17). Proceedings of the 2017 14th IAPR International Conference on Document Analysis and Recognition (ICDAR).

[39] Zhang Y., Gueguen L., Zharkov I., Zhang P., Seifert K., Kadlec B. Uber-Text: A Large-Scale Dataset for Optical Character Recognition from Street-Level Imagery. Proceedings of the SUNw: Scene Understanding Workshop—CVPR 2017.

[40] Chng C., Ding E., Liu J., Karatzas D., Chan C., Jin L., Liu Y., Sun Y., Ng C., Luo C. ICDAR2019 robust reading challenge on arbitrary-shaped text—RRC-art. Proceedings of the 2019 International Conference on Document Analysis and Recognition (ICDAR).

[41] Sun Y., Karatzas D., Chan C., Jin L., Ni Z., Chng C., Liu Y., Luo C., Ng C., Han J. ICDAR 2019 competition on large-scale street view text with partial labeling—RRC-LSVT. Proceedings of the 2019 International Conference on Document Analysis and Recognition (ICDAR).

[42] Zhang R., Yang M., Bai X., Shi B., Karatzas D., Lu S., Jawahar C., Zhou Y., Jiang Q., Song Q. ICDAR 2019 robust reading challenge on reading Chinese text on Signboard. Proceedings of the 2019 International Conference on Document Analysis and Recognition (ICDAR).

[43] Krylov I., Nosov S., Sovrasov V. Open Images V5 Text Annotation and Yet Another Mask Text Spotter. Proceedings of the Asian Conference On Machine Learning, ACML 2021.

[44] Singh A., Pang G., Toh M., Huang J., Galuba W., Hassner T. TextOCR: Towards large-scale end-to-end reasoning for arbitrary-shaped scene text. Proceedings of the 2021 IEEE/CVF Conference on Computer Vision and Pattern Recognition (CVPR).

[45] Wang K., Babenko B., Belongie S. End-to-end scene text recognition. Proceedings of the 2011 International Conference on Computer Vision.

[46] Mishra A., Alahari K., Jawahar C. Scene text recognition using higher order language priors. Proceedings of the British Machine Vision Conference 2012.

[47] Karatzas D., Shafait F., Uchida S., Iwamura M., Bigorda L., Mestre S., Mas J., Mota D., Almazan J., Heras L. ICDAR 2013 robust reading competition. Proceedings of the 2013 12th International Conference on Document Analysis and Recognition.

[48] Karatzas D., Gomez-Bigorda L., Nicolaou A., Ghosh S., Bagdanov A., Iwamura M., Matas J., Neumann L., Chandrasekhar V., Lu S. ICDAR 2015 competition on robust reading. Proceedings of the 2015 13th International Conference on Document Analysis and Recognition (ICDAR).

[49] Phan T., Shivakumara P., Tian S., Tan C. Recognizing text with perspective distortion in natural scenes. Proceedings of the 2013 IEEE International Conference on Computer Vision.

[50] Risnumawan A., Shivakumara P., Chan C., Tan C. (2014). A robust arbitrary text detection system for natural scene images. Expert Syst. Appl..

[51] Liu N., Schwartz R., Smith N. Inoculation by fine-tuning: A method for analyzing challenge datasets. Proceedings of the 2019 Conference of the North American Chapter of the Association for Computational Linguistics: Human Language Technologies, Volume 1 (Long and Short Papers).

[52] Jiang Q., Wang J., Peng D., Liu C., Jin L. Revisiting Scene Text Recognition: A Data Perspective. Proceedings of the IEEE/CVF International Conference on Computer Vision.

[53] Sheng F., Chen Z., Xu B. NRTR: A no-recurrence sequence-to-sequence model for scene text recognition. Proceedings of the 2019 International Conference on Document Analysis and Recognition (ICDAR).

[54] Cui M., Wang W., Zhang J., Wang L. Representation and correlation enhanced encoder–decoder framework for scene text recognition. Proceedings of the Document Analysis and Recognition—ICDAR 2021.

[55] Baek J., Matsui Y., Aizawa K. What if we only use real datasets for scene text recognition? Toward scene text recognition with fewer labels. Proceedings of the 2021 IEEE/CVF Conference on Computer Vision and Pattern Recognition (CVPR).

